# Supplementary material for: How personal values shape job seeker preference: A policy capturing study
Source: PLoS One. 2021 Jul 29;16(7):e0254646. doi: 10.1371/journal.pone.0254646 (PMC8320984; doi:10.1371/journal.pone.0254646)
Supplement: S1 Table — (DOCX) [file pone.0254646.s001.docx]

**S1 Table**

*High and Low Descriptors for Workplace Attributes (Based on Employer Attractiveness Scale Dimensions)*

| **Predictor** | **Low variant** | **High variant** |
| --- | --- | --- |
| Economic value | The organization offers average salary and promotion opportunities. | The organization offers above-average salary and promotion opportunities. |
| Development value | The organization has few internal programs to support employee personal and career development. | The organization has many internal programs to support employee personal and career development. |
| Interest value | The organization has a reputation for being conservative and traditional. It has well-developed policies and procedures to guide employee actions, and offers a highly predictable work environment. | The organization has a reputation for being exciting and innovative. It encourages employees to think creatively, and provides a challenging work environment where employees face new problems each day. |
| Social value | The organization has a reputation for providing a somewhat negative and unpleasant social environment for its employees. | The organization has a reputation for providing a positive and pleasant social environment for its employees. |
| Application value | The organization does not have a strong customer focus. Its primary aim is to maximize returns to shareholders. It does not believe that corporations should publicly comment on issues such as social and racial equality. | The organization has a strong customer focus. It aims to balance profitability with operating in a manner that benefits society. It publicly supports social and racial equality. |
| Environmental value | Profitability is at the heart of this organization's business model. It does not have a well-developed set of environmental policies and procedures. It does not encourage employees to adopt environmentally sustainable practices at work. | Environmental sustainability is at the heart of this organization's business model. It has a well-developed set of environmental policies and procedures. It encourages employees to adopt environmentally sustainable practices at work. |
