# Supplementary material for: How personal values shape job seeker preference: A policy capturing study
Source: PLoS One. 2021 Jul 29;16(7):e0254646. doi: 10.1371/journal.pone.0254646 (PMC8320984; doi:10.1371/journal.pone.0254646)
Supplement: S3 Table — (DOCX) [file pone.0254646.s003.docx]

**S3 Table**

*Level 2 Analyses: Job Seekers’ Personal Values Predicting Level 1 Effects*

| Effect | Coefficient | 99% CI  [LL, UL] | *SE* | *t*(397) |  |
| --- | --- | --- | --- | --- | --- |
| *L1 intercept (mean attractiveness)* | 4.22 |  | .046 | 87.06** |  |
| Self-enhancement | 0.46 | 0.32, 0.60 | .053 | 8.80** |  |
| Self-transcendent | -0.17 | -0.37, 0.03 | .078 | -2.53 |  |
| *Economic value* |  |  |  |  |  |
| Self-enhancement | -0.04 | -0.17, 0.09 | .049 | -.77 |  |
| Self-transcendent | -0.06 | -0.27, 0.15 | .080 | -.78 |  |
| *Development value* |  |  |  |  |  |
| Self-enhancement | -0.10 | -0.21, 0.01 | .044 | -2.31 |  |
| Self-transcendent | 0.06 | -0.11, 0.23 | .066 | .86 |  |
| *Interest value* |  |  |  |  |  |
| Self-enhancement | -0.04 | -0.17, 0.09 | .049 | -.91 |  |
| Self-transcendent | 0.09 | -0.10, 0.28 | .074 | 1.16 |  |
| *Social value* |  |  |  |  |  |
| Self-enhancement | -0.46 | -0.64, -0.28 | .071 | -6.50** |  |
| Self-transcendent | 0.37 | 0.13, 0.61 | .093 | 4.00** |  |
| *Application value* |  |  |  |  |  |
| Self-enhancement | -0.23 | -0.36, -0.10 | .050 | -4.47** |  |
| Self-transcendent | 0.28 | 0.07, 0.49 | .082 | 3.46** |  |
| *Environmental value* |  |  |  |  |  |
| Self-enhancement | -0.13 | -0.28, 0.02 | .060 | -2.13 |  |
| Self-transcendent | 0.58 | 0.40, 0.76 | .071 | 8.01** | |

**p* < .01, ***p* < .001. Coefficients were computed using HLM’s restricted maximum likelihood algorithm and are interpreted as average unstandardized beta coefficients. Given that the number of observations per participant was small, we computed the effects for each predictor using a random intercept and coefficient model in which all predictors were fixed, other than the one being tested. This involved re-running the model six times, once for each predictor. All Level 1 and Level 2 predictors in the model were grand mean centred. All significance tests were based on robust standard errors.
