# Supplementary figures and images for: How personal values shape job seeker preference: A policy capturing study
Source: PLoS One. 2021 Jul 29;16(7):e0254646. doi: 10.1371/journal.pone.0254646 (PMC8320984; doi:10.1371/journal.pone.0254646)

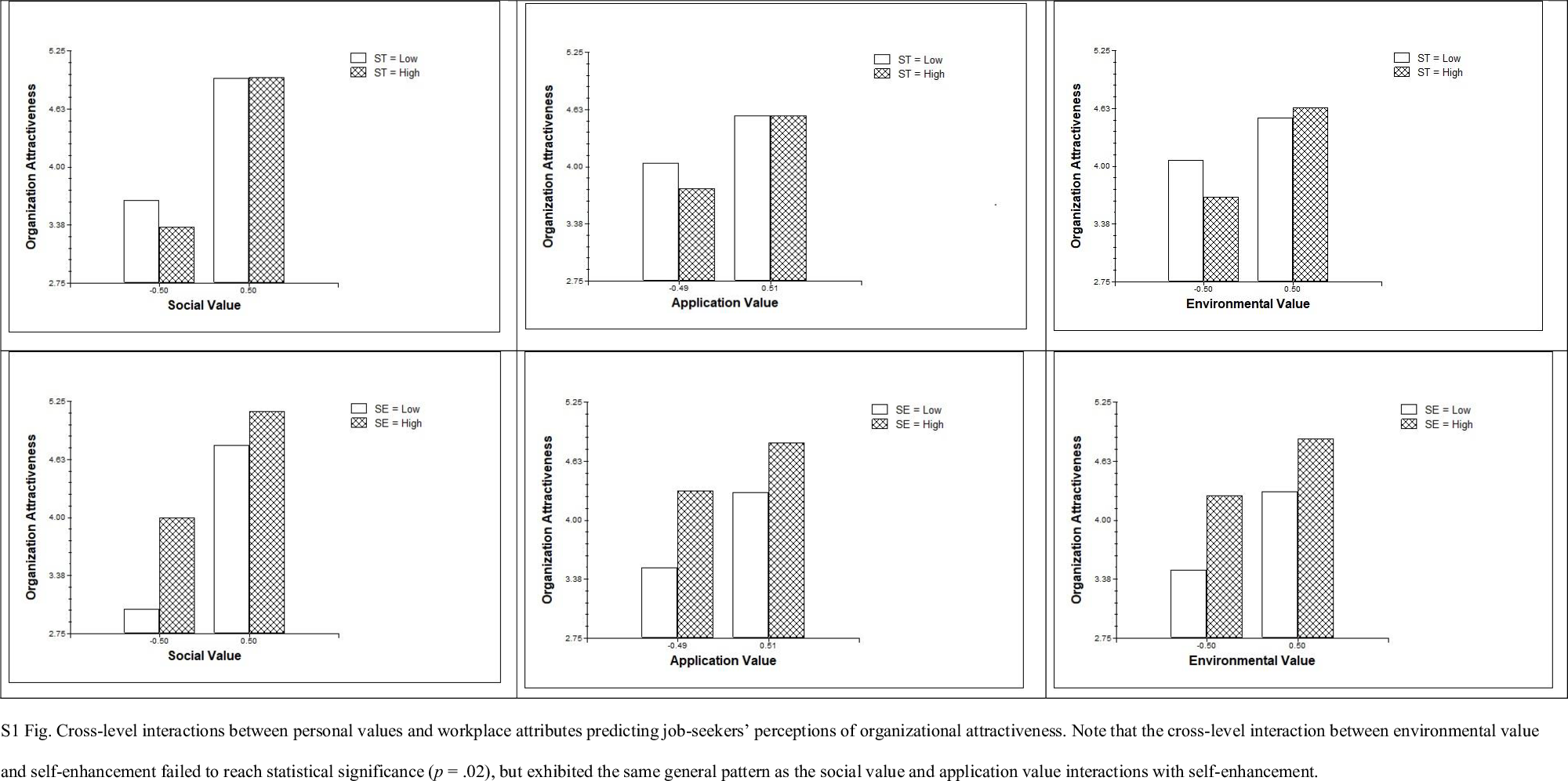

Supplement: S1 Fig — (TIF) [file pone.0254646.s004.tif]

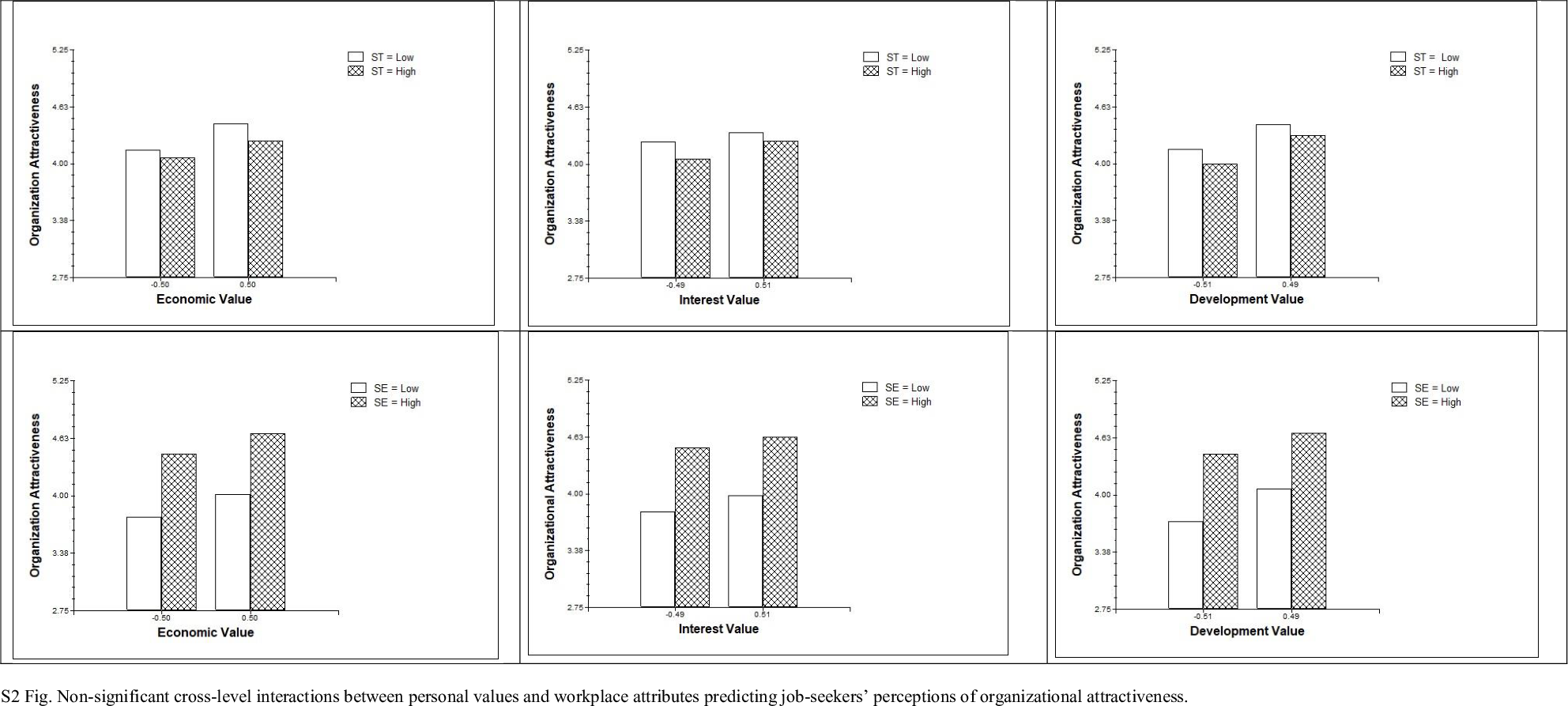

Supplement: S2 Fig — (TIF) [file pone.0254646.s005.tif]
