# Supplementary material for: How personal values shape job seeker preference: A policy capturing study
Source: PLoS One. 2021 Jul 29;16(7):e0254646. doi: 10.1371/journal.pone.0254646 (PMC8320984; doi:10.1371/journal.pone.0254646)
Supplement: Attachment — Submitted filename: Response to Reviewers.docx [file pone.0254646.s006.docx]

30 May 2021

PONE-D-21-01887
How personal values shape job seeker preference: A policy capturing study

Hi PLOS ONE editorial team,

Thank you again for your work in progressing this submission. The email dated March 26^th^ from the academic editor, Iván Barreda-Tarrazona PhD detailed the following requirements for resubmitting the paper, and we have addressed the points raised, as follows

**Reviewer #1 comments**

1. Organization attractiveness does not necessarily translate to applying for positions at a firm. What do we know about the connection between an espoused policy and likelihood of taking action on it?

Response: A paragraph discussing this has been added to the discussion section (600/26).

2. Address social desirability bias; How can we understand how the study participants might have adapted their answers to demonstrate that they had sound and socially acceptable values?

Response: Given that the survey employed an anonymous response format pressure to provide socially desirable responses should have be relatively low. In addition, to the extent that social desirability was operating, it is worth noting that this likely mimics virtue signalling in people’s real world choices, in which jobs are sometimes chosen to enhance social status and reputation concerns.

3. The reviewer found the language for the "low" condition in the scenarios to be quite negative and off putting. The reviewer suggests than saying these factors are not really present in the company or that the company didn't care about those factors might be overly negative.

Response: We have added a sentence to the Limitations and future research section to address the point you raise (588/26).

4. You asked for a fuller explanation of the impact of having only 6 individuals see the same scenario (400/64). Does the large number of scenarios in comparison to the sample size in any way inhibit your inferences?

Response; Although it is correct that only 6 participants would have seen exactly the same full scenario, 400 participants would have viewed each level (low vs high) of all of our organization attribute variables. Given that we were not investigating all possible interactions between all of the organisation attributes, the statistical power was high for all main effects and the limited number of cross-level interactions conducted.

In terms of the potential for biased estimates MLM, Maas and Jox (2005) note that in MLM the major contributor to bias/inaccuracy is the higher level sample size – in the case of our study Level 2. In their paper, they ran a series of simulations in which they varied Level 1 sample size, Level 2 sample size, and magnitude of intraclass correlations. They found that only simulations with small Level 2 samples (consisting of 50 or fewer cases) produced biased Level 2 standard errors. All other simulations, including those with Level 1 sample sizes as small as 5, produced accurate and unbiased regression coefficients, variance components and standard errors at Level 1 and Level 2.

We cite this paper and describe its findings in the revised manuscript (339/16).

Maas, C. J. M., & Hox, J. J. (2005). Sufficient sample sizes for multilevel modeling. *Methodology, 1*(3), 86-92. doi: 10.1027/1614-1881.1.3.86

5. Include in the practical implication section, specifically how firms can use the PO fit aspect of org attractiveness to reach out to the best fitting prospective applicants. Provide more information about these implications, given the paper is focused on that issue. You found that certain attributes are simply more attractive, but how can we target the best fitting applicants. I believe that the implications section of the paper needs more work to provide interesting and potentially impactful ideas.

Response; The practical implications section has been expanded with ideas on how to target the best fitting applicants and why this is important (537/25).

**Additional requirements**

1. Ensure the manuscript meets PLOS ONE's style requirements, including those for file naming.

Response; We’ve checked our submission meets PLOS ONE’s style and file naming requirements.

2. Review the reference list to ensure that it is complete and correct.

Response; We’ve checked to ensure the reference list is complete and correct.

3. Provide additional details regarding participant consent - was consent informed, were any participants minors, type of consent obtained.

Response; Additional details about consent have been added to the Procedure section (243/11).

4. Upload a copy of Figure 1 referred to on page 16 of the original submission. If the figure is no longer to be included as part of the submission, please remove all reference to it within the text.

Response; S1 Figure showing significant interactions has been updated. A second figure (S2 Figure) for non-significant interactions has also been added. If possible, we would prefer to include S2 Figure as supplementary material with a note in the article that refers to the second figure.

5. Include a copy of Tables 1, 2 and 3 referred to in on pages 14 and 16 of the original submission.

Response; These tables are included in the resubmission

6. Consider introducing more graphical analysis of all dimensions for both personal types, beyond that already present in Figure S1. The editor expected to see a figure for Hypothesis 7.

Response; A second figure for non-significant interactions has been added to the submission as supplementary material with a note in the article that refers to the second figure.

7. Consider using the same scale in the axes for all comparable figures.

Response; We have changed the figures so all axes use the same scale.

8. Specify how much we paid the Qualtrics subject sample participants and the dropout rate.

Response; This information has been added to the Procedure section (245/11).

9. Compare our sample to the general Australian population

Response; In the Participants section, we now compare our sample to the general Australian population (228/11).

10. Acknowledge possible order effects due to presenting the attributes always in the same sequence.

Response; The risk of order effects has been included in a paragraph in the Discussion section (582/25).
